# Surrogacy of intermediate endpoints for overall survival in randomized controlled trials of first-line treatment for advanced soft tissue sarcoma in the pre- and post-pazopanib era: a meta-analytic evaluation

**DOI:** 10.1186/s12885-019-5268-2

**Published:** 2019-01-11

**Authors:** Kazuhiro Tanaka, Masanori Kawano, Tatsuya Iwasaki, Ichiro Itonaga, Hiroshi Tsumura

**Affiliations:** Department of Orthopaedic Surgery, Faculty of Medicine, Oita University, 1-1 Idaigaoka, Hasama, Yufu City, Oita 879-5593 Japan

**Keywords:** Advanced soft tissue sarcoma, Randomized controlled trial, First-line treatment, Doxorubicin, Endpoint, Surrogacy

## Abstract

**Background:**

Overall survival is the true endpoint for most randomized controlled trials (RCTs) of malignant tumors, whereas progression-free survival (PFS) is considered the most reliable surrogate endpoint for overall survival (OS). The present study aimed to evaluate the correlation between surrogate endpoints and OS in randomized trials of first-line chemotherapy with doxorubicin (DOX), the standard treatment for advanced and metastatic soft tissue sarcomas (ASTS), using a meta-analytic approach.

**Methods:**

In a systematic review, we identified RCTs of first-line chemotherapy for ASTS that compared single-agent doxorubicin (DOX) with other chemotherapy regimens, and were published in English during January 1974–December 2017. A meta-analysis was performed to evaluate the efficacy of first-line treatments for ASTS. Surrogacy of the intermediate endpoints for OS was investigated using weighted linear regression analysis. Correlation strength was examined using the coefficient of determination (R^2^).

**Results:**

Twenty-seven randomized trials, comprising 6156 patients (3371 patients in the experimental arm and 2785 patients in the DOX arm) were identified. The hazard ratios for OS and PFS showed that the efficacy of treatment for ASTS was not significantly different between standard DOX and experimental treatments. The median OS was significantly prolonged in RCTs published after 2012 when pazopanib was approved for treating ASTS. The median PFS, however, did not differ significantly. The correlation between PFS and OS was moderate (R^2^ = 0.557), but better than that between OS and 3-month PFS, 6-month PFS, and response rate (R^2^ = 0.200, 0.073, and 0.278, respectively). The correlation between PFS and OS tended to be more favorable in RCTs published after 2012 (R^2^ = 0.586 and 0.459, respectively).

**Conclusions:**

The trial-level correlation between PFS and OS was only modest; it tended to be better in RCTs published after 2012. While the effective lines of chemotherapy and the introduction of new drugs prolonged OS but not PFS, PFS is a better surrogate than other intermediate endpoints in the first-line ASTS trials even in the post-pazopanib era. Although this does not negate the need for more reliable surrogate endpoints for OS.

**Electronic supplementary material:**

The online version of this article (10.1186/s12885-019-5268-2) contains supplementary material, which is available to authorized users.

## Background

Soft tissue sarcomas (STS) account for approximately 1% of all malignant tumors [1]. In total, 1529 patients with STS were registered in 2015 in Japan [2]. Approximately 50% of localized STS cases have local and/or distant recurrence, and the prognosis of patients with locally advanced and/or metastatic STS (ASTS) remains poor. The standard treatment for ASTS is systemic chemotherapy, with first-line chemotherapy regimen for ASTS being doxorubicin (DOX) [3]. The efficacy of DOX for ASTS has been demonstrated in a meta-analysis of randomized controlled trials (RCTs), and its superiority over combination chemotherapy has been confirmed [4]. However, to the best of our knowledge, none of the studies on the evaluation of endpoints of RCTs has focused on first-line chemotherapy using standard DOX for ASTS.

Overall survival (OS) is the true endpoint for most RCTs of malignant tumors, whereas progression-free survival (PFS) is considered to be the most reliable surrogate endpoint for OS [5]. For ASTS, 3- and 6-month PFS were considered appropriate endpoints in a phase II RCT [6], and have been used as primary endpoints in a phase III RCT [7].

After the approval of pazopanib (the first molecularly-targeted therapeutic agent for ASTS) in 2012 [8], trabectedin, eribulin, and olaratumab were approved for ASTS [9–11]. Although no single RCT has shown an advantage of other regimens over standard DOX, first-line olaratumab and DOX combination chemotherapy for ASTS has demonstrated superiority to DOX alone in terms of OS for the first-time [11]. The introduction of these new agents for ASTS in clinical settings and RCTs, have led to multiple lines of treatment that may prolong post-progression survival and OS. The resulting changes in the post-protocol treatment, might have led to the loss of the surrogacy of PFS and other time-to-event endpoints for OS in recent RCTs. However, the correlation between PFS or other surrogate endpoints and OS in the first-line treatment of ASTS has not been evaluated.

In this study, we conducted a meta-analysis of 27 RCTs, including recent RCTs using new agents as first-line treatment for ASTS, to investigate the surrogacy of intermediate endpoints for OS in RCTs of ASTS, and to assess the changes in the surrogacy in the post-pazopanib era.

## Methods

### Study selection

A comprehensive, systematic search of PubMed, Scopus, EBSCOhost MEDLINE, and the Cochrane Central Register of Controlled Trials was conducted in accordance with the Preferred Reporting Items for Systematic Reviews and Meta-Analyses guidelines [12]. The search algorithm followed a previously described method [13], but also included the keywords “doxorubicin” OR “adriamycin” OR “anthracycline” AND “first line” OR “first-line.” Phase II/III RCTs on first-line systemic chemotherapy for ASTS that compared single-agent DOX with other chemotherapy regimens published in English between January 1974 and December 2017 were included. RCTs of bone sarcoma, rhabdomyosarcoma and other pediatric sarcomas, Kaposi sarcoma, and gastrointestinal stromal tumors were excluded owing to the distinct biological characteristics and treatment strategies associated with those tumors. Reviews, meta-analyses, and non-RCTs were also excluded.

### Data extraction

The publication date, study phase, primary and secondary endpoints, dose of the standard arm DOX, regimen and dose of the experimental arm, presence of intention-to-treat analysis, sample size, and description of the post-protocol treatment were extracted. For OS and PFS (or time-to-progression), the medians, hazard ratios (HRs), 95% confidence intervals (CIs), and *p*-values were extracted. The response rate (RR) was defined as the proportion of assessed patients with a complete or partial response based on the criteria of each study. Data on 3-month PFS (or 12-week), 6-month (or 24-week) PFS, 1-year PFS, 1-year OS, and 2-year OS were extracted based on Kaplan-Meier estimates. When these data were not described, Kaplan-Meier curves of PFS or OS were used for the estimation as binary proportions. Data were extracted and crosschecked by two authors (K.T. and M.K.). In the case of discrepancies, a third author (T.I. or I.I.) was consulted to reach a consensus.

### Statistical analyses

Meta-analyses of pooled HRs and corresponding 95% CIs calculated for PFS and OS, or odds ratios (ORs) and corresponding 95% CIs calculated for RR, 3-month PFS, 6-month PFS, 1-year PFS, 1-year OS, and 2-year OS were performed using the Mantel-Haenszel method and an inverse variance random-effect model. Heterogeneity was quantified using a Cochrane’s Q-test and I^2^ statistics. Subgroup analyses were performed to evaluate the differences between RCTs published before and after 2012. Publication bias was evaluated using a funnel plot. Meta-analyses were performed using Review Manager software (version 5.3; Nordic Cochrane Centre, Cochrane Collaboration, Copenhagen, Denmark).

Associations between the surrogate endpoints (PFS and RR) and OS were quantified using weighted linear regression test by study sample size. Rank correlation coefficients were used to assess the association between intermediate endpoints and OS. The strength of the associations was measured using the coefficient of determination (R^2^) [14, 15]. Other statistical analyses were conducted using SAS (version 9.4; SAS institute, Cary, NC, USA). A two-sided, and *p* < 0.05 was considered statistically significant.

## Results

### Characteristics of the RCTs included in the meta-analysis

The characteristics of the eligible studies are summarized in Table 1 and (Additional file 1: Table S1 ). We identified 1479 articles. After removing duplicates, 1286 studies were further screened, and 1255 studies were excluded. The full texts of the remaining 31 articles were evaluated; two repeat publications, one study protocol, and one study on a pediatric population were excluded. A total of 27 eligible RCTs were included in the meta-analysis [7, 11, 16–40] (Additional file 2: Figure S1). The funnel plots revelaed no obvious publication bias for the RCTs analyzed in the present study (Additional file 3: Figure S2).

**Table 1:** RCT characteristics

Characteristic	RCTs before 2012 (1974–2012)		RCTs after 2012 (2013–2017)		Total RCTs	
	No. of studies, *n* (%)	No. of patients, *n* (%)	No. of studies, *n* (%)	No. of patients, *n* (%)	No. of studies, *n* (%)	No. of patients, *n* (%)
	18 (100.0)	4058 (100.0)	9 (100.0)	2098 (100.0)	27 (100.0)	6156 (100.0)
Trial phase^a^						
III	7 (38.9)	2061 (50.8)	4 (44.4)	1473 (70.2)	11 (40.7)	3534 (57.4)
II	5 (27.8)	512 (12.6)	5 (55.6)	625 (29.8)	10 (37.0)	1137 (18.5)
NS	6 (33.3)	1485 (36.6)	0 (0.0)	0 (0.0)	6 (22.2)	1485 (24.1)
Primary endpoint						
OS	0 (0.0)	0 (0.0)	2 (22.2)	1095 (52.2)	2 (7.4)	1095 (17.8)
PFS	3 (16.7)	586 (14.4)	5 (55.6)	628 (29.9)	8 (29.6)	1214 (19.7)
Other time-to-event	0 (0.0)	0 (0.0)	2 (22.2)	375 (17.9)	2 (7.4)	375 (6.1)
RR	1 (5.6)	95 (2.3)	0 (0.0)	0 (0.0)	1 (3.7)	95 (1.5)
NS	14 (77.8)	3377 (83.2)	0 (0.0)	0 (0.0)	14 (51.9)	3377 (54.9)
ITT analysis included						
No	17 (94.4)	3963 (97.7)	1 (11.1)	118 (5.6)	18 (66.7)	4081 (66.3)
Yes	1 (5.6)	95 (2.3)	8 (88.9)	1980 (94.4)	9 (33.3)	2075 (33.7)
Post-protocol treatment described						
No	10 (55.6)	2885 (71.1)	3 (33.3)	374 (17.8)	13 (48.1)	3259 (52.9)
Yes	8 (44.4)	1173 (28.9)	6 (66.7)	1724 (82.2)	14 (51.9)	2897 (47.1)

^a^Phase II/III studies were counted as phase III studies

Abbreviations: *ITT*, intention-to-treat; *No*., number; *NS*, not specified; *OS*, overall survival; *PFS*, progression-free survival; *RCT*, randomized controlled trial; *RR*, response rate

A total of 6156 patients were randomly assigned to the experimental and control DOX arms (3371 and 2785 patients, respectively). Of the 18 RCTs published before 2012 (1974–2012), in which 4058 patients were randomized, five were phase II RCTs, seven were phase III RCTs, and for six this information was not specified. Nine RCTs with 2098 patients were published after 2012 (2013–2017). Five were phase II RCTs and four were phase III RCTs. Post-protocol treatments were described in eight (44.4%) of the 18 RCTs published before 2012 and in six (66.7%) of the nine RCTs published after 2012. The use of pazopanib, trabectedin, eribulin, and olaratumab was not reported in RCTs published before 2012. However, these new agents were used in post-protocol treatments in most RCTs published after 2012.

### Differences in the median OS and PFS of the single-agent DOX arm between RCTs published before and after 2012

To evaluate recent improvements in the survival of patients with ASTS, differences in the median OS and PFS of the single-agent DOX arm of RCTs published before and after 2012 were compared.

The median PFS of the single-agent DOX arm of RCTs published before and after 2012 was 5.1 (95% CI 2.7–9.3) and 5.5 (95% CI 4.6–6.1) months, respectively (Table 2), which did not differ significantly between RCTs published before and after 2012 (*p* = 0.951). This was not surprising as all participants in the standard arm in the included RCTs had ASTS and received the same treatment, i.e. single-agent DOX. However, in RCTs published after 2012, the median OS of the standard arm was significantly prolonged (median OS before and after 2012: 9.4 [95% CI 8.4–12.0] vs. 14.5 [95% CI 13.2–27.3] months; *p* = 0.008). These findings further demonstrate that differences in the median OS and PFS in the experimental arm of RCTs published around 2012 were similar to those in the single-agent DOX arm.

**Table 2:** Difference in median OS and PFS between RCTs before and after 2012

	DOX arm			Experimental arm		
	Before 2012	After 2012	*p* value	Before 2012	After 2012	*p* value
Median OS, month (95% CI)	9.4 (8.4–12.0)	14.5 (13.2–27.3)	0.008	10.8 (9.0–11.7)	16.3 (13.3–38.9)	0.028
Median PFS, month (95% CI)	5.1 (2.7–9.3)	5.5 (4.6–6.1)	0.951	4.0 (2.0–6.8)	6.3 (2.8–8.3)	0.135

Abbreviations: *CI*, confidence interval; *DOX*, doxorubicin; *OS*, overall survival; *PFS*, progression-free survival; *RCT*, randomized controlled trial

In the experimental arm, the median OS before and after 2012 was 10.8 (95% CI 9.0–11.7) and 16.3 (95% CI 13.3–38.6) months (*p* = 0.028), respectively; the median PFS before and after 2012 was 4.0 (95% CI 2.0–6.8) and 6.3 (95% CI 2.8–8.3) months (*p* = 0.135), respectively (Table 2).

### Meta-analyses

No significant difference in OS was observed between the single-agent DOX and experimental arms (HR 0.97, 95% CI 0.90–1.04, *p* = 0.38). A subgroup analysis according to publication date also did not exhibit a significant difference in OS between the single-agent DOX and experimental arms (*p* = 0.55). The HRs for the RCTs published before and after 2012 were 0.98 (95% CI 0.91–1.06, *p* = 0.59) and 0.92 (95% CI 0.76–1.11, *p* = 0.39), respectively (Table 3, Additional file 4: Figure S3a).

**Table 3:** Summary of the meta-analyses

Endpoint	RCTs before 2012 (1974–2012)		RCTs after 2012 (2013–2017)		Subgroup analysis	Total RCTs	
	HR/OR (95% CI)	*p* value	HR/OR (95% CI)	*p* value	*p* value	HR/OR (95% CI)	*p* value
OS	0.98 (0.91–1.06)	0.59	0.92 (0.76–1.11)	0.39	0.55	0.97 (0.90–1.04)	0.38
PFS	1.04 (0.94–1.14)	0.47	0.96 (0.75–1.22)	0.74	0.56	1.02 (0.91–1.13)	0.74
RR	1.16 (0.86–1.58)	0.33	1.00 (0.58–1.74)	1.00	0.64	1.11 (0.85–1.46)	0.45
1-year OS	0.90 (0.78–1.04)	0.15	0.85 (0.65–1.12)	0.25	0.72	0.88 (0.79–0.99)	0.03
2-year OS	0.85 (0.71–1.02)	0.09	0.90 (0.65–1.25)	0.53	0.78	0.87 (0.73–1.03)	0.11
3-month PFS	1.18 (0.90–1.54)	0.23	0.99 (0.56–1.75)	0.98	0.59	1.11 (0.85–1.46)	0.43
6-month PFS	1.02 (0.81–1.28)	0.86	0.78 (0.50–1.21)	0.26	0.29	0.91 (0.73–1.15)	0.44
1-year PFS	0.90 (0.74–1.09)	0.27	0.94 (0.54–1.61)	0.81	0.88	0.88 (0.69–1.13)	0.33
2-year PFS	0.89 (0.67–1.19)	0.43	0.90 (0.63–1.30)	0.59	0.95	0.90 (0.71–1.12)	0.34

Abbreviations: *CI*, confidence interval; *HR*, hazard ratio; *OR*, odds ratio; *OS*, overall survival; *PFS*, progression-free survival; *RCT*, randomized controlled trial; *RR*, response rate

A meta-analysis of PFS revealed no significant difference between the single-agent DOX and experimental arms (HR 1.02, 95% CI 0.91–1.13, *p* = 0.74). A subgroup analysis according to publication date also demonstrated no significant difference in PFS between the single-agent DOX and experimental arms for RCTs published before (HR 1.04, 95% CI 0.94–1.14, *p* = 0.47) and after 2012 (HR 0.96, 95% CI 0.75–1.22, *p* = 0.74) (Table 3, Additional file 4: Figure S3b).

Regarding other endpoints, a meta-analysis of 3-month PFS (OR 1.11, 95% CI 0.85–1.46, *p* = 0.43), 6-month PFS (OR 0.91, 95% CI 0.73–1.15, *p* = 0.44), 1-year PFS (OR 0.88, 95% CI 0.69–1.13, *p* = 0.33), 2-year PFS (OR 0.90, 95% CI 0.71–1.12, *p* = 0.34), 2-year OS (OR 0.87, 95% CI 0.73–1.03, *p* = 0.11), and the RR (OR 1.11, 95% CI 0.85–1.46, *p* = 0.45) did not exhibit any significant differences between the single-agent DOX and experimental arms. However, 1-year OS (OR 0.88, 95% CI 0.79–0.99, *p* = 0.03) was significantly better in the experimental arm. When the analysis was restricted to RCTs published before or after 2012, none of the time-to-event endpoints or RR were significantly different between the two treatment arms (Table 3, Additional file 5: Figure S4, Additional file 6: Figure S5, Additional file 7: Figure S6, Additional file 8: Figure S7).

### Correlations between OS and the surrogate endpoints

**Fig. 1:**
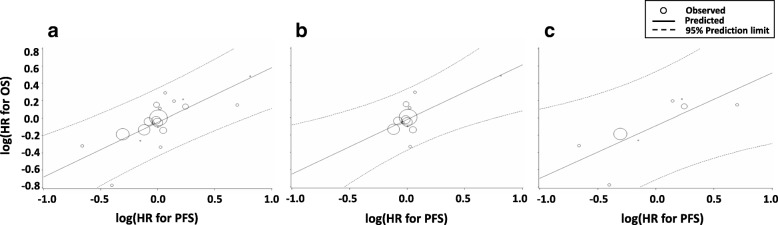
Correlation of PFS with OS. Correlation in (**a**) all RCTs, (**b**) RCTs published before 2012 (1974–2012), and (**c**) RCTs published after 2012 (2013–2017). Abbreviations: *HR* hazard ratio, *OS* overall survival, *PFS* progression-free survival, *RCT* randomized controlled trial

Overall, the correlation between OS and PFS was modest (R^2^ = 0.557, 95% CI 0.326–0.788) (Table 4, Fig. 1a-c). The Kendall rank correlation coefficient (*τ*) was 0.58. The correlation between OS and PFS in RCTs published after 2012 (R^2^ = 0.586, 95% CI 0.319–0.852; *τ* = 0.571) tended to be higher than that in those published before 2012 (R^2^ = 0 .459, 95% CI 0.216–0.774; *τ* = 0.256).

**Table 4:** Correlations between surrogate endpoints and OS

Surrogate endpoint	RCTs before 2012 (1974–2012)	RCTs after 2012 (2013–2017)	Total RCTs
	R^2^ (95% CI)	R^2^ (95% CI)	R^2^ (95% CI)
PFS	0.459 (0.216–0.774)	0.586 (0.319–0.852)	0.557 (0.326–0.788)
3-month PFS	0.103 (0.0–0.332)	0.255 (0.0–0.572)	0.200 (0.0–0.453)
6-month PFS	0.344 (0.037–0.651)	0.386 (0.064–0.708)	0.073 (0.0–0.250)
RR	0.357 (0.064–0.650)	0.228 (0.0–0.539)	0.278 (0.020–0.536)

Abbreviations: *CI*, confidence interval; *HR*, hazard ratio; *OR*, odds ratio; *OS*, overall survival; *PFS*, progression-free survival; *RCT*, randomized controlled trial; *RR*, response rate

**Fig. 2:**
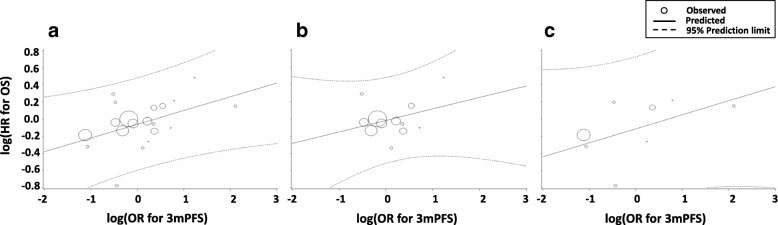
Correlation of 3-month PFS with OS. Correlation in (**a**) all RCTs, (**b**) RCTs published before 2012 (1974–2012), and (**c**) RCTs published after 2012 (2013–2017). Abbreviations: *HR* hazard ratio, *OR* odds ratio, *OS* overall survival, *3mPFS* 3-month progression-free survival, *RCT* randomized controlled trial

**Fig. 3:**
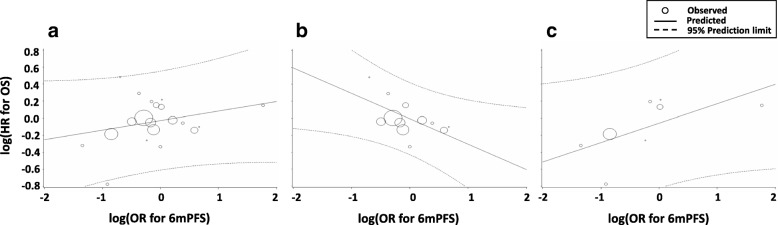
Correlation of 6-month PFS with OS. Correlation in (**a**) all RCTs, (**b**) RCTs published before 2012 (1974–2012), and (**c**) RCTs published after 2012 (2013–2017). Abbreviations: *HR* hazard ratio, *m* month, *OR* odds ratio, *OS* overall survival, *6mPFS* 6-month progression-free survival, *RCT* randomized control trial

R^2^ for associations between 3-month PFS and OS for all RCTs combined, RCTs published before 2012, and RCTs published after 2012 were 0.20 (95% CI 0.0–0.453; *τ* = 0.263), 0.103 (95% CI 0.0–0.332; *τ* = 0.030), and 0.255 (95% CI 0.0–0.572; *τ* = 0.357), respectively. Correlations between 6-month PFS and OS for all RCTs combined (R^2^ = 0.073, 95% CI 0.0–0.250; *τ* = 0.407), RCTs published before 2012 (R^2^ = 0.344, 95% CI 0.037–0.651; *τ* = 0.314), and RCTs published after 2012 (R^2^ = 0.386, 95% CI 0.064–0.708; *τ* = 0.428) were all weaker than those for PFS (Table 4, Fig. 2a-c, Fig. 3a-c).

**Fig. 4:**
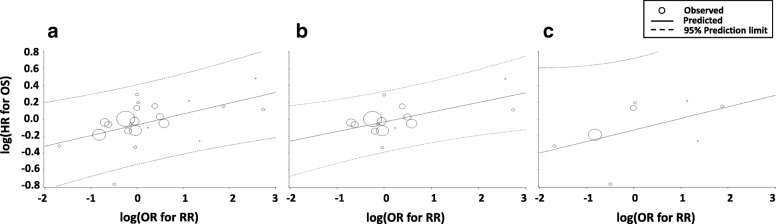
Correlation of RR with OS. Correlation in (**a**) all RCTs, (**b**) RCTs published before 2012 (1974–2012), and (**c**) RCTs published after 2012 (2013–2017). Abbreviations: *HR* hazard ratio, *OR* odds ratio, *OS* overall survival, *RCT* randomized controlled trial, *RR* response rate

Regarding the surrogacy of RR, correlations between RR and OS for all RCTs combined (R^2^ = 0.278, 95% CI 0.020–0.536; *τ* = 0.407), RCTs published before 2012 (R^2^ = 0.357, 95% CI 0.064–0.650; *τ* = 0.314), and RCTs published after 2012 (R^2^ = 0.228, 95% CI 0.0–0.539; *τ* = 0.428) were stronger than those between OS and 3- or 6-month PFS (Table 4, Fig. 4a-c).

## Discussion

In this study, a meta-analytic evaluation demonstrated that the correlation between PFS and OS was moderate in RCTs of first-line chemotherapy for ASTS, although it was better than those for other surrogate endpoints. Median OS was significantly prolonged in RCTs published in the post-pazopanib era compared to that in RCTs published before 2012, whereas median PFS was not significantly changed. Nevertheless, the correlation between PFS and OS remained modest and tended to be more favorable for RCTs published after 2012 than for those published before 2012. The results suggest that the surrogacy of intermediate endpoints for OS could not be confirmed in RCTs of first-line treatment for ASTS.

Although very few new chemotherapeutic agents for ASTS have been approved in recent decades, the approval of pazopanib, trabectedin, eribulin, and olaratumab after 2012 may have altered the treatment strategy for ASTS. In the clinical trials that were the basis for the approval of pazopanib, trabectedin, and eribulin [8–10], the drugs were mainly used in second or later lines of ASTS treatment. However, olaratumab was approved in the United States and Europe based on a phase II RCT [11], to be administered as first-line treatment in combination with DOX for ASTS. Phase II RCTs [36, 37, 39] of first-line combination therapy with DOX and trabectedin have been performed, and a phase II RCT [41] comparing pazopanib and DOX is underway. In the present study, a meta-analysis and subgroup analyses were carried out to evaluate whether the status of single-agent DOX as the standard primary treatment for ASTS has changed (before and after 2012). Our results demonstrated that even around 2012, the efficacy of the experimental arm was not significantly superior to that of single-agent DOX. Therefore, single-agent DOX remains the standard primary treatment for ASTS even in the post-pazopanib era.

We observed no significant difference in the median PFS between RCTs published before and after 2012. In contrast, the median OS was significantly prolonged in RCTs published after 2012. The use of new drugs in the post-protocol treatment in RCTs published after 2012 may have contributed to the prolonged OS despite no difference in PFS. A dissociation of PFS from OS is also possible, altering the significance of PFS as a surrogate endpoint in RCTs of ASTS.

The usefulness of 3- and 6-month PFS as surrogate endpoints has been reported in clinical trials of ASTS. Glabbeke et al. [6] examined 12 phase II studies of ASTS conducted by the European Organization for Research and Treatment of Cancer. The authors demonstrated that cutoff values of 30–56% for 6-month PFS in first-line treatment and ≥ 40% for 3-month PFS in second-line treatment should be used to determine whether a drug is effective enough to conduct phase III RCTs [6]. The advantages of these time-to-event endpoints and RR are that results are obtained quickly and with a small sample size and that they are not as affected by the post-protocol treatment as OS. Therefore, they can be used as primary endpoints in phase III RCTs [7]. However, there is major concern as to whether these intermediate endpoints are truly and strongly correlated with OS.

The surrogacy of PFS for OS has been examined in various cancers. In lung cancer, a re-analysis of six meta-analyses of 60 RCTs comprising 15,071 patients showed that for RCTs involving adjuvant chemotherapy, the strength of the correlation between disease-free survival and OS was excellent (R^2^ = 0.92) [42]. In RCTs of advanced-stage disease, the strength of the association between PFS and OS was regimen-dependent, with R^2^ values ranging between 0.89 and 0.97. The authors concluded that disease-free survival (for adjuvant chemotherapy) and PFS (for advanced-stage disease) were reliable surrogate endpoints in lung cancer [42].

In an analysis of 22 RCTs, including 16,762 cases of first-line chemotherapy for advanced-stage colorectal cancer, the correlation between PFS and OS was only moderate (ρ = 0.51 at patient level and R^2^ = 0.54 at trial level) [43]. However, Buyse et al. [5] revealed a strong correlation between PFS and OS in advanced colorectal cancer. Therefore, in first-line treatment for advanced colorectal cancer, the surrogacy of PFS and OS may have declined in recent years. This may have been affected by the lines of effective therapy for post-protocol treatment.

Only one study [13] has investigated the surrogacy of PFS and RR for OS in patients with ASTS. The trial-level surrogacy of the intermediate endpoints in 52 RCTs was investigated using the standardized beta coefficient. The correlation coefficient between the other endpoints and OS was 0.61 for PFS, 0.51 for RR, 0.27 for 3-month PFS, and 0.31 for 6-month PFS. The authors concluded that PFS and RR were appropriate surrogate endpoints for OS in RCTs of ASTS [13]. However, there are many concerns regarding the interpretation of their results as follows [44, 45]. Instead of the standard evaluation method for trial-level associations of surrogate endpoints (R^2^) simple correlation was used. Moreover, not only first-line treatment but also second- or later lines of treatment were included, and standard therapies of the trials varied widely, making the interpretation of the results difficult. Measures of variability, such as 95% CIs for the surrogacy estimates, were not shown, and details of the regimens examined in the eligible RCTs were also not presented. Furthermore, only 12 of 52 RCTs were used in the primary analysis of PFS.

In the present study, we showed that the trial-level correlation with OS was an R^2^ of 0.557 for PFS, 0.200 for 3-month PFS, 0.073 for 6-month PFS, and 0.278 for RR. The correlation between PFS and OS was modest, although the surrogacy of PFS for OS was better than those of other time-to-event endpoints and RR. Regarding the effect of new drugs, the correlation of PFS with OS was slightly higher in the RCTs published after 2012 (R^2^ = 0.586) than in those published before 2012 (R^2^ = 0.459). These results are consistent with the observation that the HRs of both OS and PFS tended to be more favorable for the experimental arm of RCTs published after 2012. Although our results are based on 21 of 27 RCTs and trial-level analyses, currently PFS was the most useful surrogate endpoint for OS in RCTs of first-line chemotherapy for ASTS.

Our study has several limitations. First, the present analysis was based on published data only and lacked individual patient data as well as patient-level surrogacy analyses. Second, patient background characteristics varied widely across the studies. Third, a number of RCTs involved patients who had received prior chemotherapy (*n* = 175; 2.8%). Thus, not all studies involved purely first-line treatments. Forth, several studies did not include a definition of the time-to-event endpoints and/or post-protocol treatment. Finally, the possibility that new drugs had been used in post-protocol treatment, even in RCTs published before 2012, could not be ruled out.

## Conclusions

In conclusion, as a surrogate endpoint in the first-line treatment of ASTS, PFS exhibited only moderate correlation with OS. Nonetheless, the surrogacy of PFS was better than those of other intermediate endpoints. Considering the rarity of ASTS and the difficulty in conducting large-scale RCTs, PFS is currently passable as a surrogate endpoint for OS in RCTs of the first-line treatment for ASTS even in the post-pazopanib era. However, more reliable surrogate endpoints for OS should be identified.

## Additional files

Additional file 1: **Table S1:** Detailed description of the RCTs included in this meta-analysis. (DOCX 29 kb)

Additional file 2: **Figure S1:** PRISMA flow diagram. (PPTX 47 kb)

Additional file 3: **Figure S2:** Funnel plot of the studies included in the meta-analysis before (a) and after (b) 2012 to evaluate the presence of publication bias. (PPTX 49 kb)

Additional file 4: **Figure S3:** Forest plot of OS (a) and PFS (b) with doxorubicin alone vs experimental chemotherapy. (PPTX 67 kb)

Additional file 5: **Figure S4:** Forest plot of 1-year (a) and 2-year (b) OS with doxorubicin alone vs experimental chemotherapy. (PPTX 68 kb)

Additional file 6: **Figure S5:** Forest plot of 3-month (a) and 6-month (b) PFS with doxorubicin alone vs experimental chemotherapy. (PPTX 73 kb)

Additional file 7: **Figure S6:** Forest plot of 1-year (a) and 2-year (b) PFS with doxorubicin alone vs experimental chemotherapy. (PPTX 69 kb)

Additional file 8: **Figure S7:** Forest plot of the response rate to doxorubicin alone vs experimental chemotherapy. (PPTX 55 kb)

## Abbreviations

ASTS: Advanced and metastatic soft tissue sarcomas
CI: Confidence interval
DOX: Doxorubicin
HR: Hazard ratio
OR: Odds ratio
OS: Overall survival
PFS: Progression-free survival
RCT: Randomized controlled trial
