# Supplementary material for: Surrogacy of intermediate endpoints for overall survival in randomized controlled trials of first-line treatment for advanced soft tissue sarcoma in the pre- and post-pazopanib era: a meta-analytic evaluation
Source: BMC Cancer. 2019 Jan 11;19:56. doi: 10.1186/s12885-019-5268-2 (PMC6330427; doi:10.1186/s12885-019-5268-2)
Supplement: Supplementary file 1 — Table S1: Detailed description of the RCTs included in this meta-analysis. (DOCX 29 kb) [file 12885_2019_5268_MOESM1_ESM.docx]

**Additional file 1: Table S1. Detailed description of the RCTs included in this meta-analysis**

| Ref. No | Author and year of publication | No. of patients in the DOX arm | No. of patients in experimental Arm | Total no. of patients | DOX Dose (mg/m^2^ per cycle) | Experimental regimen and dose (mg/m^2^ per cycle) |
| --- | --- | --- | --- | --- | --- | --- |
|  | **RCTs before 2012** | | | | | |
| 16 | Chang et al. 1976 | 18 | 15 | 33 | 60 | DOX (60) + Streptozotocin (2500) |
| 17 | Cruz et al. 1979 | 41 | 76 | 117 | 1.2 then 0.8 mg/kg weekly | 1) ActD (0.05 mg/kg) + LPAM (40 mg/Body) 2) ActD (0.05 mg/kg) + LPAM (40 mg/Body) + VCR (6 mg/Body) 3) ActD (0.05 mg/kg) + LPAM (40 mg/Body) + NSC1026 (2 g/kg) |
| 18 | Savlov et al. 1981 | 106 | 102 | 208 | 60 | Cycloleucine (200 or 300 mg/kg) |
| 19 | Schoenfeld et al. 1982 | 71 | 150 | 221 | 70 | 1) VCR (1.4) + DOX (50) + CPA (750) 2) VCR (1.4) + ActD (0.4) + CPA (750) |
| 20 | Bramwell et al. 1983 | 38 | 33 | 71 | 75 | Carminomycin (20) |
| 21 | Omura et al. 1983 | 155 | 160 | 315 | 60 | DOX (60) + DTIC (1250) |
| 22 | Muss et al. 1985 | 66 | 66 | 132 | 60 | DOX (60) + CPA (500) |
| 23 | Borden et al. 1987 | 242 | 119 | 361 | 1) 70 2) 60 then 15 weekly | DOX (60) + DTIC (1250) |
| 24 | Mouridsen et al. 1987 | 106 | 104 | 210 | 75 | Epirubicin (75) |
| 25 | Borden et al. 1990 | 176 | 171 | 347 | 70 | DOX (70) + Vindesine (3) |
| 26 | Edmonson et al. 1993 | 95 | 184 | 279 | 80 | 1) DOX (60) + IFM (7500)  2) MMC (8) + DOX (40) + CDDP (60) |
| 27 | Santoro et al. 1995 | 263 | 400 | 663 | 75 | 1) DOX (50) + IFM (5000) 2) CPA (500) + VCR (1.5) + DOX (50) + DTIC (750) |
| 28 | Nielsen et al. 1998 | 112 | 222 | 334 | 75 | Epirubicin (150 or 50 × 3 days) |
| 29 | Verweij et al. 2000 | 43 | 43 | 86 | 75 | Docetaxel (100) |
| 30 | Judson et al. 2001 | 45 | 50 | 95 | 75 | Liposomal Dox (50) |
| 31 | Lorigan et al. 2007 | 110 | 216 | 326 | 75 | IFM (3,000 × 3 days or 9,000 continuous) |
| 32 | Maurel et al. 2009 | 67 | 65 | 132 | 75 | DOX (90) + IFM (12500) |
| 33 | Demetri et al. 2012 | 42 | 86 | 128 | 75 | DOX (75) + Conatumumab (15 mg/kg) |
|  | **RCTs after 2012** | | | | | |
| 34 | Judson et al. 2014 | 228 | 227 | 455 | 75 | DOX (75) + IFM (10,000) |
| 35 | Gelderblom et al. 2014 | 39 | 79 | 118 | 75 | Brostallicin (10 then 12.5) |
| 36 | Blay et al. 2014 | 60 | 61 | 121 | 75 | Trabectedin (1.5) |
| 37 | Bui-Nguyen et al. 2015 | 43 | 90 | 133 | 75 | Trabectedin (1.3/3 h or 1.5/24 h) |
| 38 | Chawla et al. 2015 | 40 | 86 | 126 | 75 | Aldoxorubicin (350) |
| 11 | Tap et al. 2016 | 67 | 66 | 133 | 75 | DOX (75) + Olaratumab (15 × days 1 and 8) |
| 39 | Martin-Broto et al. 2016 | 60 | 55 | 115 | 75 | DOX (60) + Trabectedin (1.1) |
| 7 | Seddon et al. 2017 | 129 | 128 | 257 | 75 | Gemcitabine (675 × days 1 and 8) + Docetaxel (75) |
| 40 | Tap et al. 2017 | 323 | 317 | 640 | 75 | DOX (75) + Evofosfamide (300 × days 1 and 8) |

Abbreviations: ActD, actinomycin D; CDDP, cisplatin; CPA, cyclophosphamide; DTIC, dacarbazine; DOX, doxorubicin; IFM, ifosfamide; LPAM, melphalan; MMC, mitomycin C; RCT, randomized controlled trial; VCR, vincristine
