# Supplementary material for: Surrogacy of intermediate endpoints for overall survival in randomized controlled trials of first-line treatment for advanced soft tissue sarcoma in the pre- and post-pazopanib era: a meta-analytic evaluation
Source: BMC Cancer. 2019 Jan 11;19:56. doi: 10.1186/s12885-019-5268-2 (PMC6330427; doi:10.1186/s12885-019-5268-2)
Supplement: Supplementary file 2 — Figure S1: PRISMA flow diagram. (PPTX 47 kb) [file 12885_2019_5268_MOESM2_ESM.pptx]

## Slide 1
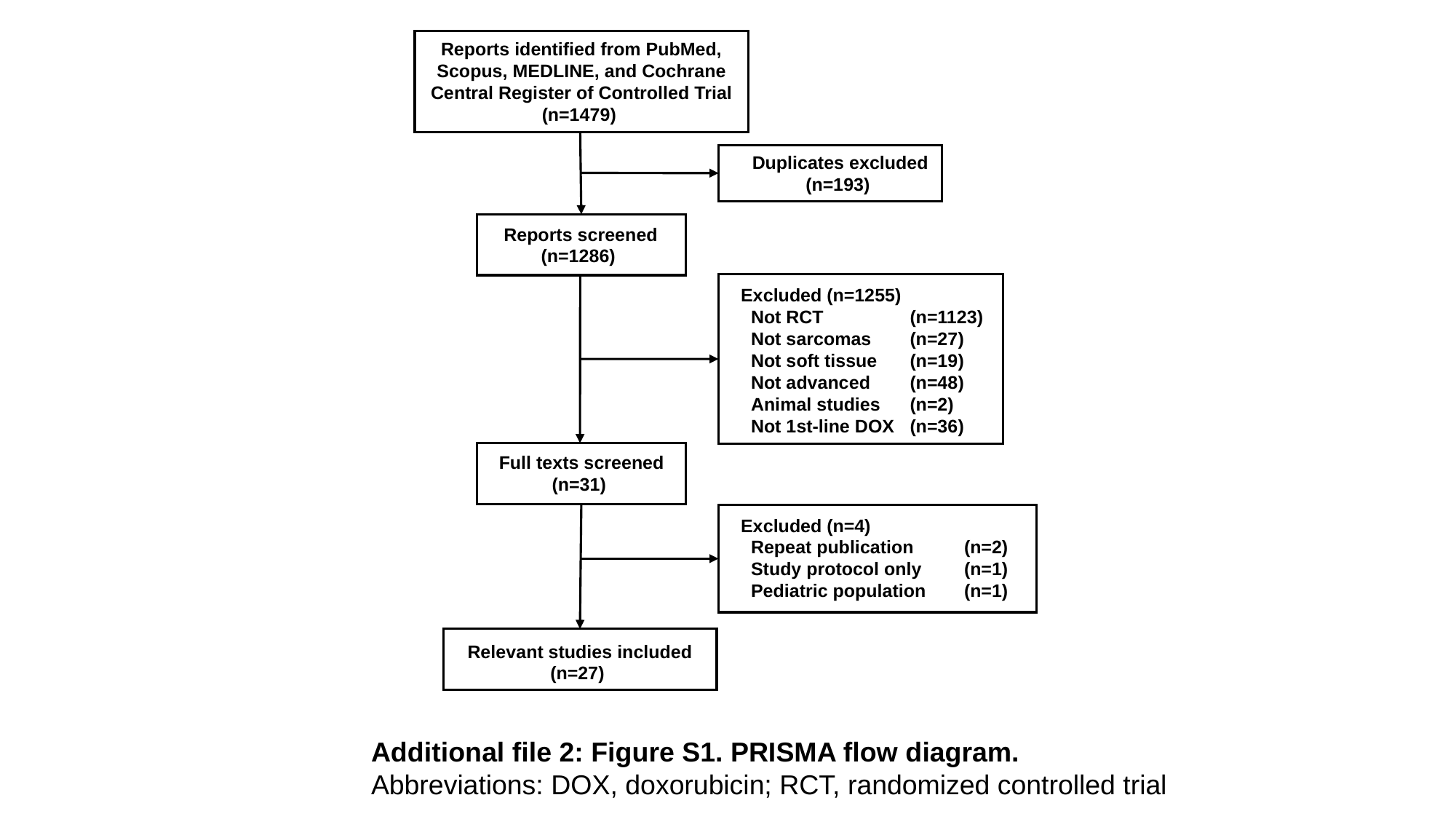

Reports identified from PubMed, Scopus, MEDLINE, and Cochrane Central Register of Controlled Trial
(n=1479)
Duplicates excluded
(n=193)
Reports screened
(n=1286)
Excluded (n=1255)
 Not RCT	(n=1123)
 Not sarcomas	(n=27)
 Not soft tissue	(n=19)
 Not advanced	(n=48)
 Animal studies	(n=2)
 Not 1st-line DOX	(n=36)
Full texts screened
(n=31)
Excluded (n=4)
 Repeat publication	 	 (n=2)
 Study protocol only	 (n=1)
 Pediatric population	 (n=1)
Relevant studies included
(n=27)
Additional file 2: Figure S1. PRISMA flow diagram.
Abbreviations: DOX, doxorubicin; RCT, randomized controlled trial
