# Supplementary material for: Surrogacy of intermediate endpoints for overall survival in randomized controlled trials of first-line treatment for advanced soft tissue sarcoma in the pre- and post-pazopanib era: a meta-analytic evaluation
Source: BMC Cancer. 2019 Jan 11;19:56. doi: 10.1186/s12885-019-5268-2 (PMC6330427; doi:10.1186/s12885-019-5268-2)
Supplement: Supplementary file 3 — Figure S2: Funnel plot of the studies included in the meta-analysis before (a) and after (b) 2012 to evaluate the presence of publication bias. (PPTX 49 kb) [file 12885_2019_5268_MOESM3_ESM.pptx]

## Slide 1
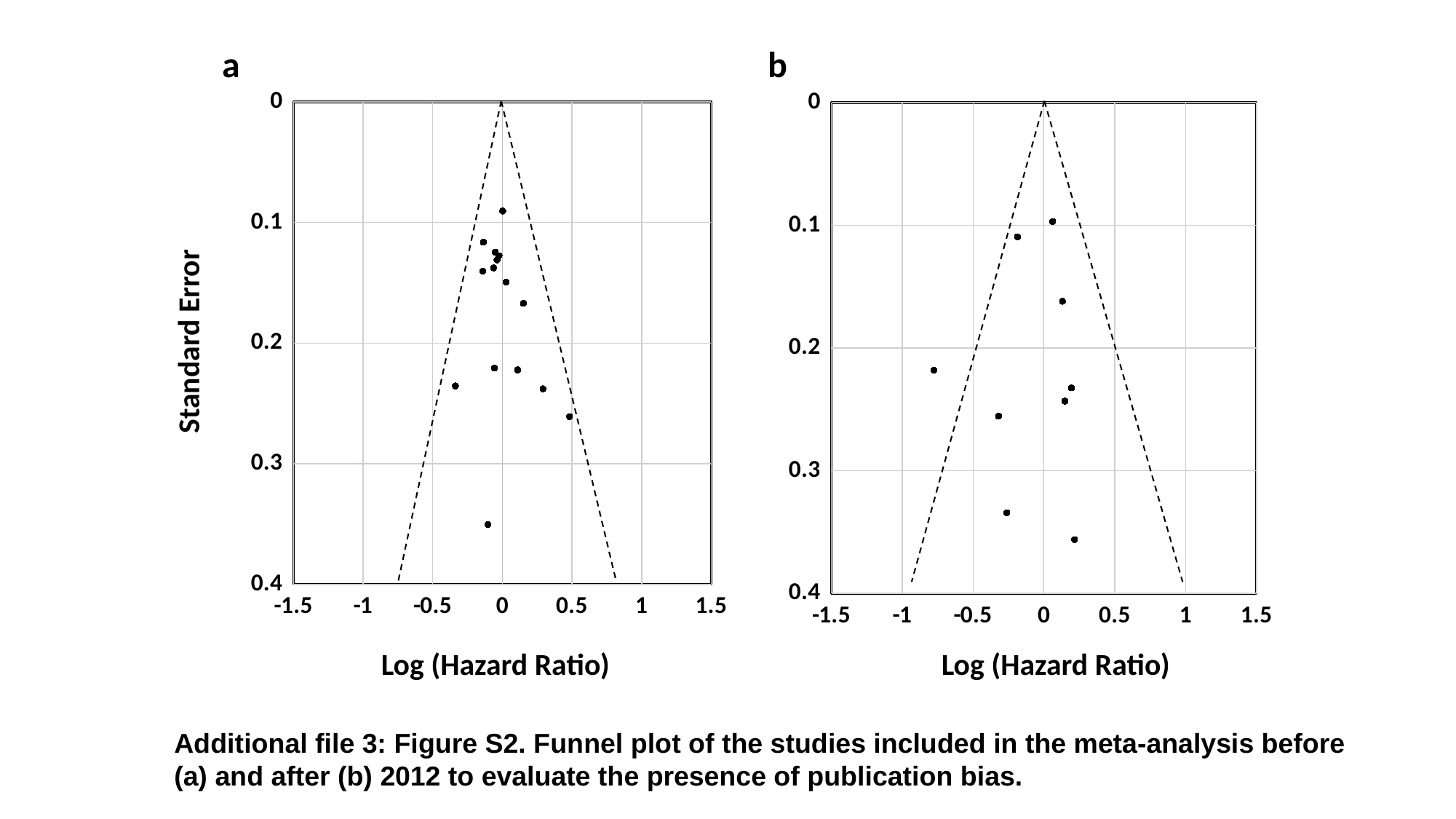

a					b
### Chart
| Category | |
|---|---|
### Chart
| Category | |
|---|---|Standard Error
Log (Hazard Ratio)
Log (Hazard Ratio)
Additional file 3: Figure S2. Funnel plot of the studies included in the meta-analysis before (a) and after (b) 2012 to evaluate the presence of publication bias.
