# Supplementary material for: Surrogacy of intermediate endpoints for overall survival in randomized controlled trials of first-line treatment for advanced soft tissue sarcoma in the pre- and post-pazopanib era: a meta-analytic evaluation
Source: BMC Cancer. 2019 Jan 11;19:56. doi: 10.1186/s12885-019-5268-2 (PMC6330427; doi:10.1186/s12885-019-5268-2)
Supplement: Supplementary file 4 — Figure S3: Forest plot of OS (a) and PFS (b) with doxorubicin alone vs experimental chemotherapy. (PPTX 67 kb) [file 12885_2019_5268_MOESM4_ESM.pptx]

## Slide 1
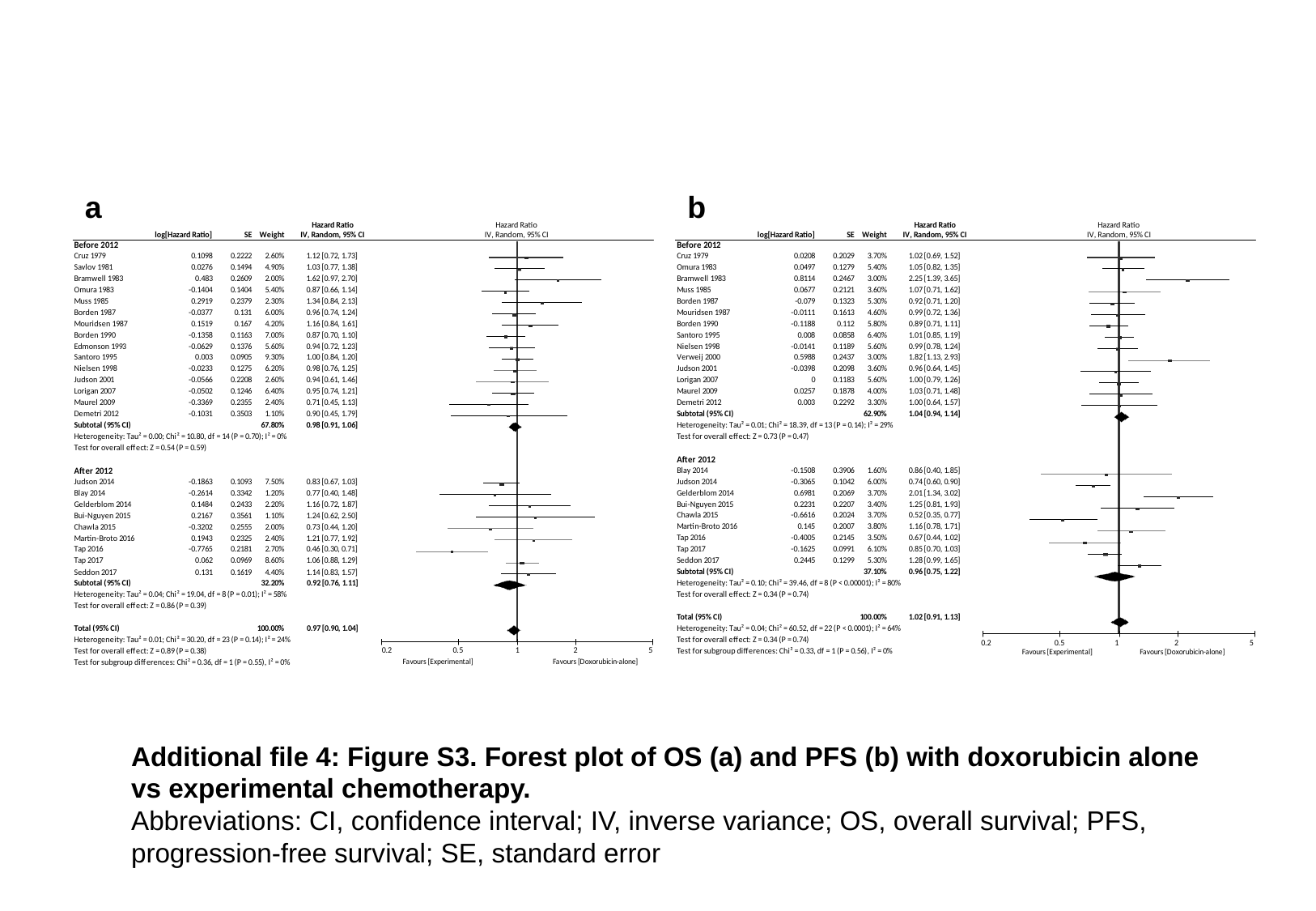

a
b
Additional file 4: Figure S3. Forest plot of OS (a) and PFS (b) with doxorubicin alone vs experimental chemotherapy.
Abbreviations: CI, confidence interval; IV, inverse variance; OS, overall survival; PFS, progression-free survival; SE, standard error
