# Supplementary material for: Surrogacy of intermediate endpoints for overall survival in randomized controlled trials of first-line treatment for advanced soft tissue sarcoma in the pre- and post-pazopanib era: a meta-analytic evaluation
Source: BMC Cancer. 2019 Jan 11;19:56. doi: 10.1186/s12885-019-5268-2 (PMC6330427; doi:10.1186/s12885-019-5268-2)
Supplement: Supplementary file 8 — Figure S7: Forest plot of the response rate to doxorubicin alone vs experimental chemotherapy. (PPTX 55 kb) [file 12885_2019_5268_MOESM8_ESM.pptx]

## Slide 1
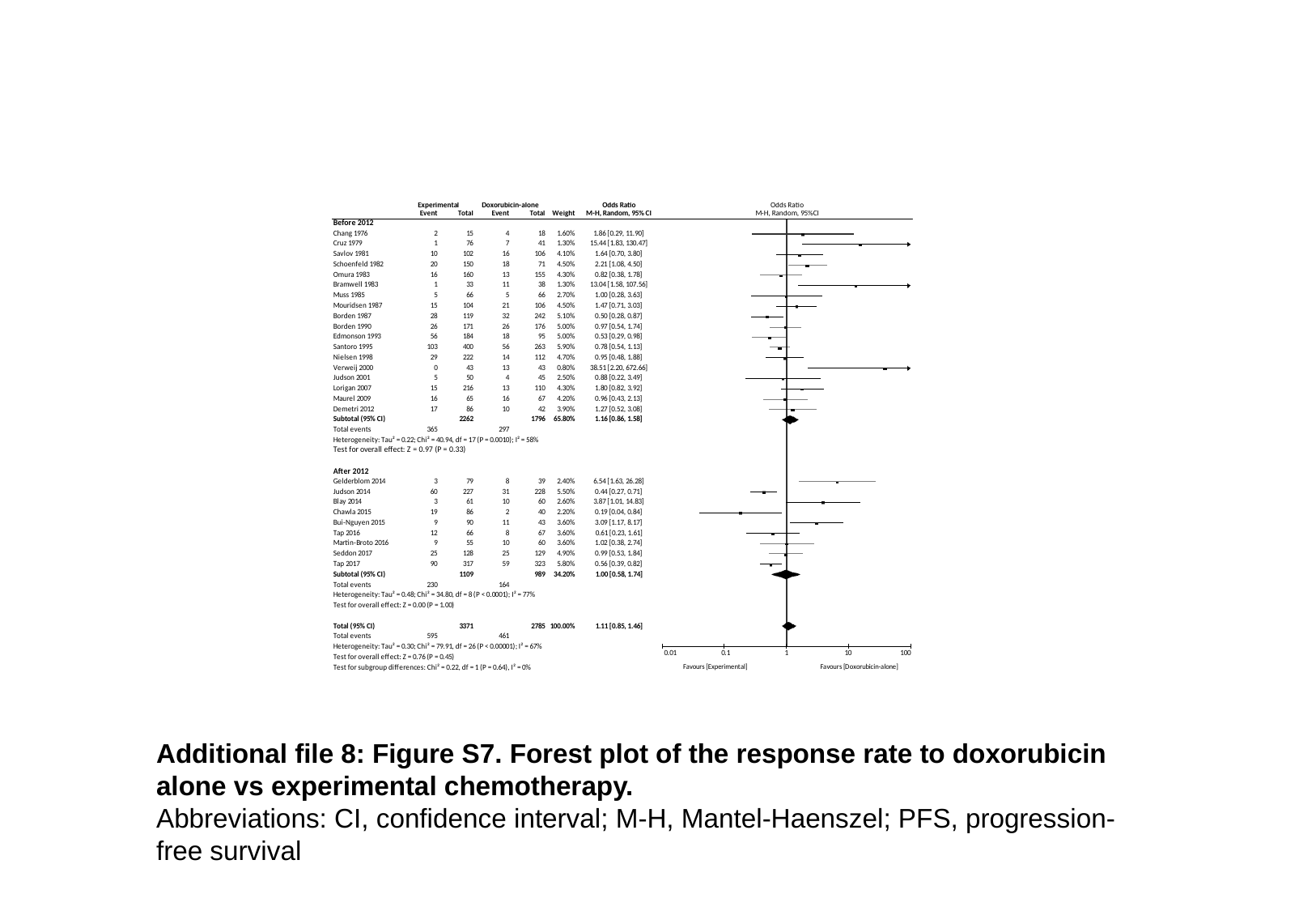

Additional file 8: Figure S7. Forest plot of the response rate to doxorubicin alone vs experimental chemotherapy.
Abbreviations: CI, confidence interval; M-H, Mantel-Haenszel; PFS, progression-free survival
